# The Bronze Age of drug checking: barriers and facilitators to implementing advanced drug checking amidst police violence and COVID-19

**DOI:** 10.1186/s12954-022-00590-z

**Published:** 2022-02-04

**Authors:** Jennifer J. Carroll, Sarah Mackin, Clare Schmidt, Michelle McKenzie, Traci C. Green

**Affiliations:** 1 Department of Sociology and Anthropology, North Carolina State University, 10 Current Drive, Campus Box 8107, Raleigh, NC 27695-8107 USA; 2 Brown University, Providence, RI USA; 3 Access, Harm Reduction, Overdose Prevention and Education (AHOPE) Syringe Exchange, Boston, MA USA; 4 The Miriam Hospital, Providence, RI USA; 5 Brandeis University, Waltham, MA USA

## Abstract

**Objectives:**

Unpredictable fluctuations in the illicit drug market increase overdose risk. Drug checking, or the use of technology to provide insight into the contents of illicit drug products, is an overdose prevention strategy with an emerging evidence base. The use of portable spectrometry devices to provide point-of-service analysis of the contents of illicit drugs been adopted by harm reduction organizations internationally but is only emerging in the United States. This study aimed to identify barriers and facilitators of implementing drug checking services with spectrometry devices in an urban harm reduction organization and syringe service program serving economically marginalized people who use drugs in Boston, Massachusetts (USA).

**Methods:**

In-vivo observations and semi-structured interviews with harm reduction staff and participants were conducted between March 2019 and December 2020. We used the consolidated framework for implementation research to identify implementation barriers and facilitators.

**Results:**

This implementation effort was facilitated by the organization’s shared culture of harm reduction—which fostered shared implementation goals and beliefs about the intervention among staff persons—its horizontal organizational structure, strong identification with the organization among staff, and strong relationships with external funders. Barriers to implementation included the technological complexity of the advanced spectroscopy devices utilized for drug checking. Program staff indicated that commercially available spectroscopy devices are powerful but not always well-suited for drug checking efforts, describing their technological capacities as “the Bronze Age of Drug Checking.” Other significant barriers include the legal ambiguity of drug checking services, disruptive and oppositional police activity, and the responses and programmatic changes demanded by the COVID-19 pandemic.

**Conclusions:**

For harm reduction organizations to be successful in efforts to implement and scale drug checking services, these critical barriers—especially regressive policing policies and prohibitive costs—need to be addressed. Future research on the impact of policy changes to reduce the criminalization of substance use or to provide explicit legal frameworks for the provision of this and other harm reduction services may be merited.

## Introduction

More than 100,000 people fatally overdosed in the United States in the 12 months ending in April 2021—a growth of 28.5% over that time [1]. Rates of fatal overdose from synthetic opioids, in particular, surged during COVID-19-related restrictions [2]. The Commonwealth of Massachusetts recorded 2394 confirmed fatalities in the 12 months ending in April 2021, reflecting a 5% increase over that time—less sharp of an increase than observed elsewhere in the United States, but an increase nonetheless, consistent with a multi-year trend of persistently high rates of fatal overdose [1]. Consensus is emerging that this surge in overdose deaths is driven by social isolation, financial instability, shrinking access to healthcare, and increased social stigma brought on by the COVID-19 pandemic [3–6]. Massachusetts has also experienced a growing HIV epidemic among populations of people who inject drugs [7], likely exacerbated by the fact that access to essential harm reduction services has declined during the pandemic. As many as 72% of syringe services programs (SSPs) across the United States limited their hours of operation when pandemic restrictions took force, 43% reduced their services, and 15% shut their doors entirely [8, 9].

Drug checking, or the use of technology to provide insight into the contents of illicit drug products, is an evidence-based strategy for overdose prevention [10–14]. Drug checking services of various kinds have been operating across Europe and North America for several decades, with an estimated 31 services operational in 20 countries by 2017 [15]. In the United States, the use and distribution of fentanyl test strips is a relatively new but increasingly common drug checking strategy employed by SSPs and other programs, typically provided alongside harm reduction materials like sterile syringes and naloxone [16]. More complex technologies, like portable spectrometry devices, have been used for drug checking in several European countries since at least the 1990s [15] yet are only now emerging as an approach to drug checking in the United States [17–19].

Recent analyses have explored the technical capacities of portable spectrometry devices, including limits of detection and the interpretation of spectrometry outputs [20, 21]. Research on the participant uptake and analytical results of drug checking services through the use of chemical reagents (a strategy most often used for testing stimulants and other psychoactive substances commonly considered “club drugs”) or portable spectroscopy devices at electronic music events has been carried out in European countries including Italy [22], Portugal [23] Slovenia [24], and the United Kingdom [25]. Importantly, recent qualitative studies conducted in Vancouver, Canada, have explored the perceptions of drug checking participants and providers as well as drivers of and barriers to the uptake of drug checking services with advanced spectrometry devices [26–31]. One of these studies utilized an implementation science framework to conduct a pre-implementation study informed by interviews with potential service users (people who use and/or distribute drugs) [31]. A multi-site investigation conducted in three U.S. cities undertook a similar pre-implementation study with harm reduction and public health stakeholders poised to provide drug checking services to local participants [32]. To the best of our knowledge, post-implementation outcomes of harm reduction-based drug checking services utilizing portable spectrometry devices have not yet been systematically evaluated.

The purpose of this study is to describe the implementation of drug checking services with portable spectrometry devices as part of direct service provision at an SSP in Boston, Massachusetts and to identify barriers and facilitators shaping implementation efforts in this context.

## Methods

Data presented here were collected as part of a multi-site assessment of novel harm reduction strategies in the Commonwealth of Massachusetts (United States). This study focuses on one SSP where we sought to evaluate the implementation of a new drug checking program intended to increase participant engagement at the SSP and facilitate linkage to low-barrier medication treatment through a partnering clinic. Data collection for that evaluation took place between March 2019 and December 2020. We conducted three site visits to the SSP during this time (March 2019 for initial planning, June 2019, and June 2020) to observe the delivery of drug checking services and informally discuss the successes and challenges of the program with staff. Unstructured, descriptive fieldnotes were taken to capture information about context, events, statements, and processes as they occurred [33].

As part of the evaluation process, we conducted semi-structured, qualitative interviews with 8 SSP staff (many of these repeat interviews and joint interviews) and with 4 SSP participants. Interview guides included several a priori domains including perceptions of the local drug market, participant needs, satisfaction with receiving services or service delivery, and the lessons that SSP staff were taking from their experiences. Staff were recruited by members of the research team. Participants were referred by staff and self-referred through flyers posted onsite. The interview process began immediately after recruitment, and informed consent was carried out by a research team member prior to the interview. All interviewees must have been 18 years old or older and have been capable of providing informed consent. SSP participants were offered $25 in cash for completing an interview.

Between site visits, and during the first few months of the COVID-19 pandemic when in-person visits were not possible, regular calls were scheduled with SSP leadership. Prior to the pandemic, these check-in calls were informal and were used to discuss logistical matters relating to the collection of process measures and to gain general insights on how the program was progressing, including: number of syringes distributed, number of patients referred to buprenorphine treatment for opioid use disorder, and the number of unique drug checking services provided. After March 2020, when the pandemic began, the research team conducted remote interviews with program staff to explore what new challenges they were facing, how they adapted to those challenges, and whether they were satisfied with the resulting changes.

We used the Consolidated Framework for Implementation Research (CFIR) to analyze field notes and interview transcripts [34]. The CFIR includes 37 constructs across 5 domains of program implementation: intervention characteristics; inner setting (or characteristics of the organization where implementation takes place); outer setting (such as external policy environment and relationships with other entities); characteristics of individuals involved in the implementation; and implementation process [34]. All data were deductively coded across these constructs and domains. Because the CFIR does not account for client-centered outcomes or client experiences [35], we also inductively coded data for emergent themes related to perceptions of and satisfaction with drug checking services that were not identified by the CFIR. Findings from these analyses were then discussed by the entire research team to isolate key barriers, facilitators, and events that influenced the implementation of this new drug checking program, which we describe below.

This protocol, including minor amendments to the protocol such as masking and social distancing for COVID-19 mitigation while in-person data collection continued during the pandemic, was approved by the Institutional Review Board at Rhode Island Hospital.

## Results

Despite many internal and external changes during the study period, the barriers and facilitators of implementation remained largely constant and were found across all 5 CFIR domains (see Table 1). In the sections below, we first describe the basic structure of the intervention (“Initial intervention plan” section). We then discuss in more detail the implementation facilitators and barriers identified across each of the five domains: Characteristics of the Intervention (“Characteristics of the intervention” section), Outer Setting (“Outer setting” section), Inner Setting (“Inner setting and implementation” section), Characteristics of Individuals (“Characteristics of individuals” section), and Implementation Process (“Implementation process” section).

**Table 1:** Barriers and facilitators to the implementation of advanced drug checking services between March 2019 and December 2020

CFIR domains	Facilitators	Barriers
Characteristics of the intervention	The technology works right out of the box. Use of the devices is adaptable to various locations and settings, with the right preparation. Trialability is high. This intervention is easy to scale up or down rapidly. The relative advantage of using advanced technology for drug checking (compared to fentanyl test strips or no drug checking at all) is apparent.	Drug checking is a new use case for this technology that presents many challenges. A large amount of time and energy is needed for troubleshooting. The reliability and/or interpretability of spectrometry results is sometimes poor, which requires experience, additional training, and skill to overcome. The technology is expensive and the cost of confirmatory testing is an ongoing and large expense.
Outer setting	Financial support was provided by a third party organizations for acquiring new technology. Mutual aid came from a collective of harm reduction organizations providing drug checking with advanced technology. Support came from community partners with professional expertise in spectroscopy devices and interpretation. Meaningful customer support came from device manufacturers.	Current state policies do not adequately clarify the legality of activities that are part of the drug checking process. Aggressive law enforcement activities put program staff and participants at risk of harm and make the provision of essential harm reduction services unsafe for all. The COVID-19 pandemic and concomitant social distancing measures required resources be diverted and limited capacity to engage with participants in a safe and private manner.
Inner setting	A culture of harm reduction is shared. An understanding of the goals and relative benefits of offering drug checking services is shared. A largely horizontal organizational structure allows for flexibility and a (desirable) redundancy of skills. The intervention is very compatible with the culture of the organization.	The organization is largely supported by grants that provide restricted funds. Drug checking consumes a meaningful quantity of disposable materials, from proprietary swabs and pads to aluminum foil and nitrile gloves. Keeping a steady supply of these resources may, at times, be challenging.
Characteristics of Individuals	Staff have a strong sense of self-efficacy. Staff maintain strong personal identification with the SSP. Interest among participants was very high, and the intervention clearly responded to an unmet need.	Some staff, especially those with lived experience of substance use, may have prior criminal records, which puts them at greater risk should law enforcement interfere with staff or services.
Process	Staff frequently reflected on service delivery and incorporated lessons learned into implementation strategies. The SSP is flexible enough to use trial and error in determining appropriate procedures and logistics. Engagement with participants provides staff with proof of concept that drug checking is an effective intervention.	Many challenges faced by the SSP could not have reasonably been anticipated prior to implementation.

### Initial intervention plan

Prior to the conceptualization of drug checking services in this setting, the SSP was already in close collaboration with a not-for-profit healthcare for the homeless program (HCHP). The central goals of that collaboration included increased access to on-demand medication treatment for opioid use disorder, improved linkage to HIV care, and active outreach to re-connect with individuals who have become disconnected from care. SSP staff were able to refer participants directly to the HCHP, and these two programs often conducted mobile outreach in tandem, with harm reduction services offered from the SSP van and a vehicle operated by the HCHP offering wound care, HIV, STI, and Hepatitis C testing, and same day initiation of buprenorphine or naltrexone for opioid use disorder. The provision of harm reduction services by the SSP is the primary method of engagement between the HCHP and people at risk of overdose; thus, the drug checking program was conceived as a strategy to increase participant engagement and attract new participants to the SSP, thereby increasing the number of individuals receiving testing and treatment from the HCHP. The initial plan for implementation also consisted of making drug checking available both in the SSP’s brick-and-mortar location and on joint outreach efforts with the HCHP.

The 22-month observation period for this study began in March 2019 with the unboxing of the SSP’s first portable spectrometry device: an MX908 (908 Devices, Boston, MA) high-pressure mass spectrometer (HPMS) that scans trace amounts of a sample (here, drug residue from trash surrendered by SSP participants, like cookers or baggies) and provides automated results. About a year later, the SSP acquired a Bruker ALPHA II (Bruker Corporation, Billerica, MA) Fourier-transform infrared spectroscopy device (FTIR). The FTIR differs from the HPMS in that it analyzes visible samples of drug residue, not trace samples. In contrast to the text-based output of the HPMS (the device’s screen literally displays the word “fentanyl” when fentanyl is detected), the FTIR output consists of a line graph indicating the intensity of light reflected from the sample at different wavelengths, which requires a large electronic library of known substances and a trained operator to interpret.

Over the course of the observation period, the SSP’s implementation plan evolved: first, to relocate drug checking away from the HCHP program vehicle due to legal and logistical concerns; second, to add an FTIR device to the SSP’s technological arsenal; and third, to restrict the use of portable spectroscopy devices to the SSP’s fixed location. Alongside these programmatic changes, the SSP was also affected by major police operations in their area of service in the last half of 2019 and the COVID-19 pandemic from March 2020 through the end of the study period. All the while, SSP staff sought ways to connect participants more effectively with drug checking services and to provide seamless connections between drug checking services, harm reduction services, and clinical care. We further discuss these adaptations (and the successes and challenges that accompanied them) in the sections below.

### Characteristics of the intervention

#### Facilitators of drug checking services in the characteristics of the intervention

The primary facilitators of implementation in this domain included the fact that drug checking services were an internally motivated effort developed by like-minded harm reductionists as well as the perceived quality and validity of the evidence supporting drug checking as a harm reduction strategy. An SSP manager recalls the motivation to implement drug checking evolving organically, with external funders equally motivated to support that effort: “*Well I was like hey, we should do this thing… and [the foundation] was like fuck yeah, we wanna do this.”* That excitement was fueled not only by emergent research about drug checking as a harm reduction strategy, but also by the intuition that the demand for drug checking services would be high—both among their existing population and among new populations who had not previously been attracted by their services. One staff member reflected, “*It’s just a tool to engage, to get people to start talking to me, right? And once you get them talking to you, you never know where it’s gonna go.”* Other staff persons expressed excitement about the capacity to leverage drug checking services to “*[reach] more of the stimulant population…people who-people who use methamphetamine, and people who use cocaine…*” An SSP manager was equally optimistic that drug checking services would attract people “*who are sniffing”* or who “*maybe will start injecting at some point but have not yet…The ‘Unicorn’ population…they’re really fucking hard [for SSPs] to find.*” In more precise terms, SSP leadership was optimistic that drug checking would facilitate engagement with populations who use stimulants and who smoke, sniff, or ingest their drugs orally rather than inject and linking PWUD to evidence-based care, services, and safe supplies. This belief and the shared sense of ownership in the intervention fostered enthusiasm and commitment for its successful implementation.

#### Barriers to drug checking services related to characteristics of the intervention

The sheer technical complexity of the intervention proved to be a major barrier to the implementation of drug checking services. In one sense, staff described the HPMS as *“relatively simple to use.”* Insert sample. Hit button. Read results. At the same time, getting that process to run smoothly, without interruption or error, proved perennially challenging. In the words of a staff person who helped take the HPMS out with the SSPs mobile services:

*One of the head sales guys [from 908 Devices], he’s like, “I sell this to the military. Like, they use this in Iraq.” …I’m thinking to myself, “[Outdoors in] the South End [of Boston] isn’t as bad as Iraq indoors, why the fuck isn’t this machine working right now?”*

In a word, drug checking constituted a new use case that the technology was not always prepared for, giving what staff described as *“a rocky start”* to the program.

The HPMS posed myriad challenges. The machine would frequently alarm for errors of ambiguous origin, especially in unpredictable outreach environments*.* The device frequently returned implausible results; one odd pattern led to the discovery that it returned false positives for hydrocodone when scanning cocaine residue. With frequent use, the device was prone to overheating and the device’s core would become clogged with sample residue, requiring lengthy cleaning processes between scans. One SSP manager observed:

*The reality is the technology has not caught up…For [mobile outreach], ideally, we would have something that was cheaper. Like a Toughbook. That’s maybe that size or less. And that’s super accurate and can tell you percentages. And what the cut is. And it doesn’t require a lot of kinda like finagling to get a good read on it…We are in, like, the Bronze Age of drug checking.*

The manufacturer provided firmware updates to resolve some issues, but the SSP ultimately decided to scale back use of the HPMS on mobile outreach, where they often faced unpredictable technical challenges and where staff and participants alike became frustrated by inconsistent results. Instead, the HPMS was kept in the drop-in center where the environment was better controlled and where staff had access to the tools they needed to troubleshoot as complications arose.

Over the first year of implementation (April 2019-March 2020), it became increasingly clear that the HPMS device, alone, could not support the service they hoped to provide: “*It breaks down all the time, fuckin’ always like spending time fixing it or calling [customer support]. And then the shit it gives us is wild, like wild results.”* Based on the collective experience of other harm reduction organizations running drug checking programs in North America (discussed further in Outer Setting, below) and prior research on the utility of other advanced technologies [20], SSP leadership decided to purchase the FTIR, which can detect multiple substances in a single sample, including “cut,” and offer some insight about substance concentrations.

The FTIR required significant training to use, made feasible by partnerships the SSP maintains with regional academic and healthcare institutions. During one observation, a Masters-level chemist hired by a university lab had come to the drop-in center to help train staff on appropriate methods for interpreting FTIR results. Reflecting on their experiences with the two devices, one staff member mused:

*You need a visible sample for the FTIR…In order to do trace amounts, the [HPMS] is helpful… Also, you can’t look at the FTIR or spectra and be like, “Oh, I can clearly tell that this is what’s—” It takes a lot of expertise…I’m just learning that there is no one machine that's perfect for this.*

Funding from the Massachusetts Department of Public Health, in collaboration with a local university, allowed the SSP to send most of the samples they check to a more advanced lab for confirmatory testing through gold standard testing methods, such as gas chromatography mass spectrometry (GCMS). Confirmation takes up to two weeks, which limits the ability to provide that feedback to participants in real-time. However, it has enabled SSP staff to cross reference results and refine their interpretation skills when providing point-of-care drug checking services.

### Outer setting

#### Facilitators of drug checking services in the outer setting

Third-party organizations providing financial and technical support to the SSP throughout the study period were lynchpin facilitators of this drug checking service. A private foundation provided the funds necessary to purchase the $65,000 HPMS, a $6500 support package, and the materials to test ($6/test). That same foundation subsequently became aware of the difficulties staff were facing with the HPMS and, again, enabled the purchase of the FTIR (costing approximately $40,000). The purchase of even one of these devices, let alone both, required sizeable funding that the SSP did not have. The drug checking program would have been all but infeasible without this support.

Further, the SSP joined a virtual, international network of harm reduction researchers and organizations (from the United States, Canada, Mexico, Colombia, New Zealand, England, and more) who are also implementing drug checking services with portable spectrometry devices. It was the experiences of these network members that first inspired the SSP purchase an FTIR. An SSP manager described this peer-network as essential for all involved: *“[In case] we have questions about things, or like there’s opportunity for us to do [a] kind of like mutual aid society with harm reduction programs that are doing this.”* The SSP regularly used the support services provided by the manufacturers of the two spectroscopy devices in their possession. However, when the needs of the SSP staff exceeded the capacities of those services, the output interpretation and trouble-shooting expertise collectively held by members of the network proved invaluable.

#### Barriers to drug checking services in the outer setting

While the challenges posed by the complexity of the spectroscopy devices were, in the words of one staff person, “*wild*,” characteristics of the external policy environment (specifically, legal ambiguity, police activity, and COVID-19) presented the most significant barriers to the implementation of this drug checking service. First, like many similar efforts across the United States and elsewhere, this drug checking program came into existence and continues to operate in a “*legal grey area.*” Though the original project proposal envisioned drug checking services co-located in the HCHP mobile clinic, some clinicians voiced concerns about workflow challenges in the already cramped vehicle and discomfort working in proximity to illicit drugs—even in trace amounts found on surrendered trash. As a result, drug checking was “*never [carried out] on the [HCHP] van”* as intended but *“only on the [SSP] van.”* In addition, SSP staff sometimes refrained from discussing drug checking services with partnering clinicians. One outreach staff person described:

*We’re not terribly open about it…If it’s a provider [on outreach] that we know really well, we’re happy to talk to them about it, show ‘em how it works…[But] if some random doc from [the local hospital] called us out, we probably wouldn’t be super public that we’re still actively doing [drug checking].*

This low profile protected SSP staff and participants from possible police interference while on outreach and the HCHP from legal liabilities (whether real or perceived) that might follow from association with a legally ambiguous drug checking program.

Several months into implementation, SSP leadership learned they had underestimated the risks of that “*legal grey area”* when local police leadership, with whom the SSP had historically had a positive relationship, confirmed that drug checking with trace samples of illicit substances could give any officer probable cause for arrest. An SSP manager recalled:

*We went down [a major street] where everybody was openly injecting. We had the [HPMS] out and we were doing a ton of testing. But…I found out that it was illegal. I was like, oh shit... [After] a conversation with our deputy superintendent… I was like, maybe we need to be a lot more discreet about having it on the street.*

This changed the calculus for integrating drug checking services with mobile outreach and discouraged the SSP from using the HPMS outside their drop in center.

In August 2019, SSP operations were further disrupted by police activities in Boston’s South End. In response to a physical altercation between a pedestrian and an off-duty prison guard, local police initiated “Operation Clean Sweep,” during which a large police contingent forcibly cleared the neighborhood where the SSP operates. This resulted in 34 arrests for a variety of vice and quality of life crimes and the displacement of numerous structurally vulnerable residents [36]. Though the formal sweep lasted only one day, SSP staff described a pattern of police violence that lasted for months:

*It’s been fucking terrible….[One] guy took [a woman] to the ground while she had a needle in her neck. She came in here sobbing. This is [the] shit that we are hearing constantly….Treating people like shit. Calling them junkies. Telling them to get the fuck off our [the SSPs] front steps. It was an all-out fucking war on drug users.*

Staff further reported that these police activities hindered their ability to locate participants and engage them in care and services:

*It was just so constant, like cops—you could hear constantly over loudspeakers screaming at people, people being told to move, so many reports coming in from our people about abuse by [officers]...It was really difficult to find people. It was really difficult to engage with people.*

During this time, SSP leadership also reported that individual officers voiced their intention to disrupt with harm reduction services—and even arrest SSP staff—if they were perceived as “interfering” with police efforts to keep the area clear.

**Fig. 1:**
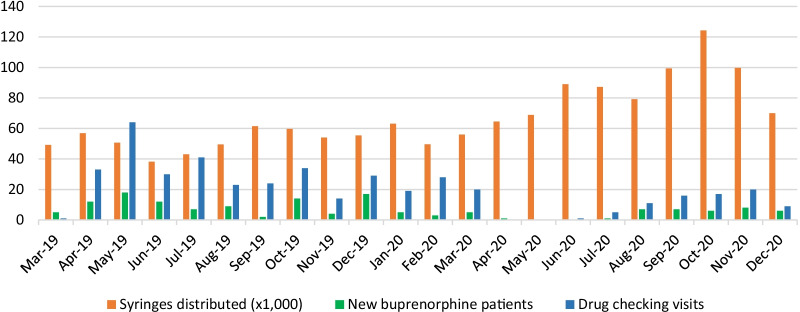
Syringes distributed (× 1000), drug checking visits served, and new buprenorphine patients enrolled at a large syringe service program in Boston, MA, March 2019 to December 2020

Operation Clean Sweep effectively ended drug checking outside the drop-in center, with staff reporting they *“[had] not taken the [HPMS] out of the drop-in…or taken it out with any joint clinical outreach since Operation Clean Sweep.”* As the SSP kept their drop-in center open and connected with participants through mobile outreach as best they could, the number of client contacts and the quantity of supplies distributed (injection equipment, naloxone kits, etc.) remained relatively constant. The quantity of drug checking services provided, however, dropped by half (Fig. 1). Between the arrival of the HPMS in April 2019 to Operation Clean Sweep in August 2019, the SSP had been fulfilling an average of 42 drug checking requests each month. In August 2019, they fulfilled only 23 requests. The frequency of drug checking remained similarly low until March 2020, which marked the beginning of COVID-19 pandemic restrictions in the United States.

Whereas Operation Clean Sweep reduced the delivery of drug checking services by half, the COVID-19 pandemic ground these services to a halt. The SSP ceased all activities that required hand-to-hand transfers including self-serve distribution of injection equipment (replaced by pre-assembled supply kits) and drug checking trash items that participants gave to outreach workers. The drop-in center was also closed, and all remaining activities were moved outside to a small courtyard or to mobile outreach, which was scaled up dramatically as the pandemic began. Fortunately, COVID-19 coincided with a change in police behavior as well. Staff reported that *“by the time COVID hit, [aggressive police activity in the neighborhood] had basically dissipated.”* Nevertheless, SSP leadership remained uncomfortable providing drug checking anywhere besides the drop-in center, which was now temporarily closed.

By the fourth quarter of 2020, the SSP had settled into a “new normal” of pandemic-restricted services, and staff had become fully trained on the use of the FTIR device in the meantime. The drop-in center remained closed, but drug checking services had returned in a new form. Specifically, staff would collect trash from participants during mobile outreach, bring those items to the drop-in center for analysis, and deliver any findings to participants in person or over the phone in the subsequent days. SSP leadership anticipated this protocol would remain in place until the drop-in center could be reopened.

### Inner setting and implementation

#### Facilitators of drug checking services in the Inner Setting

Characteristics of the inner setting of the SSP, including the culture of the organization, shared goals of SSP staff, and the relatively horizontal structure of the organization, were major implementation facilitators. The most significant of these was the culture of harm reduction shared across the organization. For many, a commitment to harm reduction represented a commitment to simple, positive, and person-centered principles. One staff person reflected, *“I just show up and love people and help them pretty much. So, whatever that means in the moment, like that's what [harm reduction] means.”* This unified orientation toward harm reduction principles served several functions. It anchored the daily work of the SSP to a set of shared values, which, in turn, clarified program priorities and unified the staff around shared goals. As one staff person described, “*[W]e have to share fundamental values. Like we have to kind of like understand the pillars of harm reduction.”* In the context of drug checking services, the common culture of harm reduction also clarified the relative priority of the intervention and generated common implementation goals:

*When someone comes in [for drug checking], that's an awesome opportunity to meet somebody literally where the fuck they're at, right? They are right there in front of you in that moment, they want you to test their shit, because they're going to use it, and you have a fucking machine that's going to give that information…That's the first step.*

Thus, staff perceived drug checking not only as congruent with the harm reduction culture and mission of the organization but also as a powerful opportunity to advance those goals and provide more effective prevention services.

The SSP’s horizontal organizational structure was also an important facilitator of implementation. Many staff persons described themselves as *“on the same level, in that we’re hired as Public Health Advocates.”* This provided redundancy the SSP needed to remain flexible when faced with disruptions or acute participant needs requiring immediate attention. At the same time, staff perceived *“a lot of leeway where we find our own niche,”* which left all staff persons feeling equally empowered to participate in the drug checking program (*“Most of us use the [HPMS] machine regularly.”*) and facilitated implementation during chaotic shifts as a result of that redundancy (*“We all wear multiple hats around here”*). This, in turn, fueled the staff’s engagement with and enthusiasm for the program. As a participant of the SSP noted about the drug checking program, *“They’re very good at what they do here. The compassion they have…It still amazes me. And you don’t see a lot of burnout with them.”*

#### Barriers of drug checking services in the inner setting

One barrier to implementation was identified in the inner setting of the SSP: the finances necessary to implement drug checking with advanced technology like HPMS and FTIR devices outstripped the resources of the SSP. An SSP manager mused, “*Where the fuck is the money gonna come from?…The state won’t even fuckin’ buy fentanyl test strips at this point…there’s no fuckin’ way they would buy a $65,000 machine.”* Maintenance of those machines is also expensive, and regular use of these devices requires consumables ranging from cotton swabs and paper cups to proprietary, single use testing strips. The SSP could not have implemented drug checking services on its own. As discussed in “Facilitators of drug checking services in the outer setting,” above, this challenge was largely overcome with the help of a private foundation. By the end of this study, regular costs like machine maintenance, subscriptions to electronic reference libraries for the FTIR, confirmatory testing, and consumable supplies like gloves, fentanyl test strips, and cleaning supplies were supported through funding from the Massachusetts Department of Public Health and other private foundations.

### Characteristics of Individuals

#### Facilitators of drug checking services in the characteristics of individuals involved

Many implementation facilitators were observed within this domain, including staff’s strong belief in the intervention, sense of self-efficacy, personal identification with the organization, and readiness to embrace and sustain the intervention. One staff person expressed his commitment as follows:

*I want to just like shout it from the rooftops, like, “Hey, we have this amazing machine that will definitely tell you if there’s fentanyl in your shit, if there’s fentanyl in that shit. We’ll be able to 100 percent tell you there’s fentanyl in it.”*

Though identification with the organization was already high, the implementation of drug checking services strengthened that identification still further. Staff excitedly declared, *“We're-we're the vanguards. Like, we're tryin' to figure this [drug checking] shit out. It's messy, but it's good.”* Most staff expressed a combination of pride and gratitude when reflecting on the opportunity to work at this SSP and participate in drug checking.

#### Barriers to drug checking services in the characteristics of individuals involved

One barrier to implementation was observed among the individuals involved: that certain staff persons with previous criminal records may have been more at risk of police harassment or other criminal-legal consequences when providing drug checking services. An SSP manager reflected, *“Like to offer a semi-illegal service to people, like, sucks. 'Cause, I mean, we're putting staff and participants at risk.*” The manager further said that “*[staff] were fairly aware up front what the kind of legal grey area was”* when drug checking services began. In interviews, SSP staff and leadership acknowledged open questions about the legality of the service and voiced concern for the staff’s wellbeing as they carried out services that were potentially criminalized.

### Implementation process

#### Facilitators of drug checking services in the Implementation process

The SSP effectively facilitated the implementation of drug checking services by ensuring that time was taken to plan the implementation strategy, adjust that strategy as circumstances demanded, and reflect on what was learned along the way. Even before the technical challenges with the HPMS were fully understood, SSP staff made a number of adjustments to their protocol. An SSP manager recalled:

*It’s really trial and error. Like, the machine is relatively simple to use. But like you do have to have a space. And you have to have a little bit of time and some organization so you're not cross-contaminating things…it took some prep…it was trial and error.*

This trial and error also characterized the experience of shifting away from point-of-care drug checking services during the COVID-19 pandemic. One outreach staff said, *“[We] get back to people by calling them, or the next time they come to the van, we’ll give them their results…It’s a start right?”* Ensuring privacy over the phone or when sharing drug checking results during mobile outreach required similar adaptability from the staff.

SSP staff also put significant energy into reflecting on their process and identifying lessons learned. For instance, acquiring the FTIR made the task of providing real-time drug checking services technologically onerous, forcing the realization that “*You can get really burnt out doing just [drug checking with the FTIR] all the time*.” By working with university partners, staff also quickly recognized that expertise in chemistry, alone, could not guarantee effective drug checking; interpreting FTIR results also required good knowledge and intuition about drug effects and the illicit drug supply. One staff person described chemistry expertise, harm reduction expertise, and personal experience with substance use as the *“triangle perfecto of who makes a really good drug checker.*” Further, the need for experience and expertise for interpreting FTIR results made the peer-network of drug checking organizations more important than ever. According to an SSP manager, *“You need a community to learn this shit…We could not do this on our own.”*

Finally, staff were able to observe and reflect upon the most noteworthy impacts that drug checking services were having for their participants. One staff person who regularly operated the HPMS and FTIR devices recalled instances in which drug checking information prompted participants to say, *“Oh, I'm not gonna inject it. I'm gonna booty bump it, or I'm gonna snort it, I'm going to smoke it.”* She reflected, *“That just reinforces how important it is.”* An SSP manager reported the intervention was reaching drug distributors as well:

*[W]e've had dealers come in here and test their shit. And we've let them know what cuts and what percentages…And people have gone back [to their batch] and maybe, thrown a little bit more [cutting agent]. That's really exciting because…that has a trickle-down effect for more people.*

SSP staff also described encounters perceived to be proof of concept that drug checking offers an effective pathway into treatment. An SSP manager spoke of a participant who had fallen out of care at the HCHP after testing positive for HIV. Outreach staff found her upset about her HIV status and “*on a tear”* (i.e. bingeing cocaine)*.* She rejected staff’s attempts to engage her until she was offered drug checking services. The manager recalled:

*She’s like, “You mean to tell me you’re going to test my shit?” I’m like, “Yeah!” She went back to the van, did drug checking, and they started talking. That actually led to her talking to the doctor. Sitting down with the doc and working on her HIV care. Like, I could not make up a more perfect story.*

Other stories recounted as proof of drug checking’s impact as an engagement tool included housed and employed persons who use drugs intermittently seeking drug checking for stimulant and opioid drugs they planned to use. For example, one occasional user was surprised to learn that his “heroin” contained fentanyl. The client subsequently developed a prevention strategy with staff that involved changing his intended route of administration. Though not every participant interaction for drug checking illustrated such direct impacts, these instances reinforced how much the intervention could offer and maintained staff commitment to persevering in offering those services.

#### Barriers to drug checking services in the implementation process

Though these barriers are more appropriately categorized in the characteristics of the intervention and the outer setting domains, respectively, the technological challenges with the drug checking devices and the tumultuous external environment—first police violence then a global pandemic—indirectly affected the SSP’s implementation process. Constant adaptations to unanticipated contingencies limited the ability to create a long-term plan, assess the fidelity of their program to that plan, and evaluate the efficacy of their approach. Quantitative measures of program output, such as the quantity of harm reduction supplies distributed and the number of drug checking service requests received, were measured over the course of this study (see Fig. 1). At the same time, recognizing whether those numbers represent failure due to interference or success despite the odds is impossible. When asked for their personal assessment, one staff person reflected, “*I think we’re doing it. I really do. Like…over a longer period of time, we can see progress getting made.”* Thus, despite many challenges, staff remained motivated by the shared belief that the drug checking services brought them closer to achieving the harm reduction goals of individual empowerment and safer use.

## Discussion

This study is, to the best of our knowledge, the first systematic investigation of the barriers and facilitators of implementing drug checking services with portable spectroscopy devices, including both HPMS and FTIR devices, in a harm reduction setting. This study followed an urban SSP over 22 months, during which time staff persevered through technological challenges, legal ambiguity, police violence, and a global pandemic. While changing external conditions dampened this intervention’s impact, the nature of what constituted barriers and facilitators did not fundamentally change through the course of events (see Table 1). In brief, the implementation was facilitated by the shared culture of harm reduction at the SSP (which in turn fostered shared implementation goals and beliefs about the intervention among staff persons), horizontal organizational structures, staff identification with the SSP, strong relationships with external funders and peer organizations tackling the same challenges, and the ongoing integration of lessons learned to the implementation strategy over time. Barriers to implementation included the technological complexity of the HPMS and FTIR devices, challenging legal and policy environments (including confrontational police activity), pandemic restrictions, and challenges to the implementation process because of these external disruptions.

Perhaps our most significant finding is that all of this SSP’s success in implementing drug checking was achieved *in spite of*, rather than thanks to, the legal and policy environment in which it operates. The COVID-19 pandemic made drug checking practically impossible, but only after systematic police violence against participants and threats against staff had already curtailed provision of this harm reduction service. Indeed, it is likely that drug checking could have persisted during the pandemic absent the very real risk of police interference. This finding mirrors that of qualitative pre-implementation studies of drug checking services in Canada and the United States, which found the criminalization and stigmatization of substance use to be the most likely barriers to implementation [31, 32].

There is clear, scholarly consensus that law enforcement and criminal justice responses to substance use exacerbate—and sometimes even generate—the individual-level harms of substance use [37–42]. This study demonstrates in striking terms how essential harm reduction organizations are equally vulnerable to the misguided, punitive responses so often directed at the people they serve. Policymakers have not sufficiently protected harm reduction efforts from state violence. Stricter, more reliable, more enforceable protections against the impacts of drug criminalization are desperately needed. De-criminalization of substance use remains an effective strategy for resolving these and other related concerns [43, 44].

Another barrier was the ambiguous legal status of drug checking activities. Some states have enacted laws that unambiguously legalize the distribution of fentanyl test strips but fail to clarify the legal status of using advanced technologies like portable spectroscopy devices for the same purpose (examples include Illinois Public Act 101–0356 and North Carolina § 90–113.22). As of May, 2021, at least 10 U.S. states had legislation pending that would create a legal framework for some drug checking activities [45]. In light of the fact that the U.S. Department of Health and Human Services (HHS) explicitly endorsed community drug checking by permitting grantees to spend federal funds on fentanyl test strips [46], any absence of a legal framework for making use of those technologies, including in Massachusetts where this study took place, is particularly glaring. This study demonstrates the importance of providing a clear legal framework for community drug checking with any technology.

This study also demonstrates that available technologies fail to fully meet the needs of drug checking programs. As legal avenues for drug checking services expand, additional resources at the local, state, and federal levels should be directed towards the improvement of portable spectrometry devices and other technologies that might be useful for drug checking. The organization included in this study, and the broader drug checking peer-network in which they participate, has already made enormous advancements in the use of these devices for community drug checking [47]. Dedicated and well-resourced efforts to leverage that expertise and advance existing technologies could quickly bring drug checking out of its proverbial “Bronze Age.”

The cost of acquiring and using this technology is one that many harm reduction organizations cannot manage. The 26-billion-dollar global settlement from opioid litigation will soon be distributed to cities and counties across the United States, creating unprecedented opportunity to invest in high-demand, high-impact interventions like community drug checking. As states are developing their global settlement spending plans [48], numerous experts have urged state leaders to dedicate that funding toward the support of essential harm reduction services, including drug checking [49]. Further, as portable spectroscopy devices perform the same function as fentanyl test strips, but do so more expansively, there is a strong rationale to expand the use of federal funds to include both fentanyl test strips and other drug checking equipment. These instruments are often purchased for forensic reasons and maintained by law enforcement and forensic laboratories. Support for their purchase, use, and extension to public health and harm reduction realms would be a way for HHS to explicitly support community drug checking.

Finally, this study highlights the unique capacity of harm reduction organizations to successfully implement drug checking services, as the culture of harm reduction, itself, facilitates implementation across numerous CFIR constructs [34]. Harm reduction takes what Dan Bigg called “any positive change” as the primary goal of any interaction. Organizations need not appoint formal implementation leaders or elevate champions for implementation when each staff person is already a champion for harm reduction. Organizations also need not impose artificial rewards or incentive structures to ensure program implementation when staff perceives their work as its own reward. A strong culture of harm reduction unifies program leadership and staff around common values, and those values translate into shared goals for and commitments to drug checking services. While it may be possible to implement successful and effective drug checking services in other contexts (medical clinics, health departments, behavioral health services, and so on), organizations without a strong harm reduction ethos may face additional barriers to implementation.

These results are subject to certain limitations. Our findings may not be applicable to non-urban settings or organizations. Our findings may also have limited applicability beyond the United States or in regions in which criminal drug policies and cultures of law enforcement are dissimilar from those studied here. The organization included in this study operates independently and maintains generally positive relationships with local leadership. These findings may not apply where harm reduction lacks meaningful support from social, clinical, and political leadership.

## Conclusions

Harm reduction organizations appear to be appropriate settings for the implementation of drug checking services. For harm reduction organizations to be successful, critical barriers need to be addressed, especially ambiguous legal frameworks, regressive policing policies, prohibitive costs, and needs for technological support. Future research on policy change to reduce the criminalization of substance use or to provide explicit legal frameworks for the provision of this and other harm reduction services may be merited. The flexibility and adaptability of community drug checking services, even during pandemic circumstances, suggest promise for improving knowledge of opaque drug markets for safer consumption, expanding overdose prevention, and better linking PWUD to care.
